# Job stressors in relation to burnout and compromised sleep among academic physicians in India

**DOI:** 10.3233/WOR-230383

**Published:** 2024-06-07

**Authors:** Karen Belkić, Neeti Rustagi

**Affiliations:** a Department of Oncology/Pathology, Karolinska Institute, Stockholm, Sweden; b Department of Medical Radiation Physics and Nuclear Medicine, Karolinska University Hospital, Stockholm, Sweden; c Claremont Graduate University, School of Community and Global Health, Claremont, CA, USA; d Institute for Health Promotion and Disease Prevention Research, University of Southern California School of Medicine, Los Angeles, CA, USA; e Department of Community Medicine and Family Medicine, All India Institute of Medical Sciences, Jodhpur, India

**Keywords:** Working conditions, physicians, hospitals, teaching, clinical medicine, occupational health, burnout, psychological, sleep

## Abstract

**BACKGROUND:**

Burnout among physicians, especially in the academic setting, is an urgent concern, with adequate sleep one of the key focal points.

**OBJECTIVE:**

To identify job stressors contributing to burnout and compromised sleep among academic physicians, using a comprehensive, theory-based instrument, the Occupational Stressor Index (OSI), whose specific form was created ‘for physicians by physicians’.

**METHODS:**

This parallel mixed-methods cross-sectional investigation was conducted among 109 physicians employed in a public teaching hospital, Jodhpur, India. Work conditions were evaluated by the physician-specific OSI (part I). The Copenhagen Burnout Index and Pittsburgh Sleep Quality Index (PSQI) were the outcome instruments (part II). Seventy-six physicians completed parts I and II.

**RESULTS:**

The physicians were from wide-ranging specialties, and 82% of the cohort were residents. Mean total OSI scores were 87.4±8.1, with unit-change yielding adjusted odds-ratios (95% confidence-intervals) for personal (1.10 (1.02–1.18)) and work-related burnout (1.12 (1.04–1.22)), and PSQI (1.09 (1.01–1.17)). Significant multivariable associations with burnout and/or sleep indices included: working 7 days/week, lacking work-free vacation, insufficient rest breaks, interruptions, many patients in intensive-care, no separate time for non-clinical duties, pressure to publish, injury/suicide attempts of colleagues/staff, performing pointless tasks. The latter were described as administrative/clerical. Lacking genuine rest breaks was mainly patient-related, further compromised by emergency work and lacking separate time for non-clinical duties. Long workhours and exhausting schedule were cited as most difficult parts of work, while reducing workhours, improving work schedule, and hiring more staff most frequently recommended.

**CONCLUSION:**

Specific working conditions potentially contributory to burnout and compromised sleep among physicians working in academic medicine are identified using a methodologically-rigorous, in-depth approach. These findings inform evidence-based interventions aimed at preserving physician mental health and work capacity.

## 1 Introduction

The long-standing global shortage in the health workforce, exacerbated by the coronavirus disease 2019 (COVID-19) pandemic, leading to major collapses in healthcare systems, spurred a clarion call, on the basis of the systematic review of Poon et al. [1], to identify specific working conditions contributory to burnout among health care workers. Burnout among physicians is of particular concern [2–8]. Other systematic reviews have indicated that individually focused and organizational measures can potentially reduce and help prevent physician burnout [8–11]. However, the evidence is considered to be of low quality, with the need for organizational interventions to “better focus on reduction of specific stressors” [10] (p.2).

A critical stumbling block in these efforts has been the adequate assessment of the complex work environments faced by physicians. Whereas an integrative, theory-based approach is needed, the methodology should also be occupation-specific, especially to pinpoint potentially modifiable job stressors. The Occupational Stressor Index (OSI) fulfills these two, frequently discordant requirements [12].

Having been developed within the framework of participatory action research [13], the physician-specific OSI reflects hands-on experience and was presented to colleagues as ‘for physicians, by physicians’. At the same time, the OSI is a comprehensive work-stressor model with its foundation in cognitive ergonomics and brain research [12, 14]. The OSI provides an index of the total stressor load of a given work environment. It incorporates key dimensions of the generic work stressor models, such as ‘high demands’ analyzing these in terms of time and allocation of mental resources, with a separate aspect addressing extrinsic time pressure. As its name implies, through the OSI the emphasis is upon ‘stressors’, i.e. the objective characteristics of a given set of work conditions [12, 14]. All specific OSI’s (e.g. for physicians, nurses, professional drivers, teachers), as well as the generic OSI, are numerically and theoretically compatible. Irrespective of the occupation, the overall burden of job stressors is reflected in the total OSI score. The OSI also can be used for in-depth analysis of the nature of that burden [14] akin to theory-guided observational approaches that assess the mental structure of job tasks [15]. Yet, being questionnaire-based, the OSI does not require on-the-job analysis, although its precision can be further improved thereby.

The OSI has been used in the clinical setting, including among physicians who faced a heavy burden of job stressors and who were at risk or suffered from burnout, sleep disorders or other stress-related health challenges [12, 14, 16, 17]. Based upon clinical experience, a total OSI score above 88 was identified as the cutpoint above which urgent intervention was needed. Interventions that lowered total OSI were consistently associated with clinical improvement [14, 18, 19].

The OSI for physicians has also been applied in research investigations [4, 18, 20–23]. Most recently, significant multivariable associations between the total OSI score and burnout were found among 22 physicians working in a dedicated outpatient respiratory center for patients with suspected or confirmed COVID-19 [23]. Numerous potentially modifiable contributory stressors were identified via the physician-specific OSI, including infrequent, disrupted rest breaks, interruptions during work, heavy outpatient load, including responsibility for transferring patients who needed emergency care, other duties besides clinical work and being contacted outside workhours about patients.

Several methodologies have been used to assess burnout [24–28], whose original definition encompassed emotional exhaustion, depersonalization, and diminished personal accomplishment [24]. The Copenhagen Burnout Inventory (CBI), evaluates three components: (A) personal burnout, as physical and emotional exhaustion not directly related to work; (B) work-related burnout, as how much the former is associated with work per se; and (C) how much physical and emotional exhaustion is associated with working with patients or other clients [27]. The CBI is especially well suited to physicians, has been applied in a many health care settings internationally [4, 27–31], and will be employed in the present study.

The emotional burden faced by physicians and other health providers has also been linked to poorer sleep [32]. Physiologic studies concordantly indicate that burnout is associated with suppressed slow wave sleep, poorer sleep efficiency and longer sleep latency. These sleep disturbances may contribute to the fatigue associated with burnout [33, 34]. Moreover, polysomnographic studies reveal that sleep (particularly Stage 2 and Rapid Eye Movement Stage) is compromised by night shift work [33, 35]. The duties of physicians may require night shift work, which has been repeatedly reported to deleteriously impact sleep [36–38]. In addressing the way forward regarding physician burnout, adequate sleep was highlighted as a key issue [39]. The present study will address sleep as an outcome, using the Pittsburgh Sleep Quality Index (PSQI), a well-established generic measure, with strong reliability and validity in a wide variety of settings [40].

As much as quantitative analysis is vital in investigating the relation between job stressors and the outcome measures, in their study applying the physician-specific, nurse-specific and generic OSI vis-à-vis burnout, Khoa et al. [21] recommended that future investigations include qualitative analysis. A mixed methods approach [41, 42] has shown itself to be fruitful in occupational health research, including studies of burnout among health professionals. This was seen, for example, in the investigation of Shinan-Altman, Werner and Cohen [43], as well as in the study carried out in the ambulatory care setting, in which insights from open-ended queries complemented the quantitative data provided by the specific OSI’s [23].

The academic medical setting is especially burdensome, associated with burnout and other untoward manifestations [3, 6, 8, 14, 19, 44, 45]. In the study of physicians working at a public teaching hospital in India just before the COVID outbreak, the initial focus was on the relation between the total OSI scores and burnout. Therein, the clinically-defined total OSI cutpoint, 88, warranting urgent intervention was corroborated by over 3-fold and 6-fold odds ratios, respectively, of personal and work-related burnout [4]. The present paper takes the next key steps. Namely, we will investigate the relation between individual stressors identified from the OSI vis-à-vis burnout among academic physicians. Sleep adequacy, as a critical concomitant will also be examined. Moreover, the physicians responded to open-ended, work-related queries. We will hereby perform a comprehensive analysis of all these available quantitative and qualitative data, using a mixed-methods approach [42]. This multi-faceted exploration can directly inform intervention strategies aimed at maintaining the work capacity and protecting the mental well-being of academic physicians in the critical post-pandemic period.

## 2 Methods

### 2.1 Study design and setting, description of the participants

This cross-sectional investigation was carried out among physicians working in an academic medical institution in Jodhpur, India. Altogether 305 physicians who performed clinical work were eligible to participate in this study, which was approved by the Regional Ethics Committee. There were no monetary incentives whatsoever; participation was entirely voluntary and was carried out during the physician’s free time during the latter part of 2018 through 2019. All but 12 of these physicians were contacted in person. The aim of the study was described, namely to enhance the health and well-being of physicians, by examining job stressors, burnout and sleep quality. Each contacted physician was asked to give informed consent with confidentiality fully guaranteed. The physicians were assured that they were free to withdraw from the study without any consequences. A total of 125 physicians (43% of those who were contacted) gave their consent and were subsequently contacted via email. The two-part web-based survey was provided through an encrypted link. Each participant received at least three reminders to complete both parts.

### 2.2 Objective information from the academic medical institute records

Some information was available about the working conditions at the Institute. This was gleaned from Institute records and policy.

### 2.3 Quantitative data from the questionnaire

Initial queries were included about age and gender in both parts I and II. Part I contained the physician-specific OSI questionnaire. The OSI for physicians questionnaire and the OSI score sheet are available at Supplemental Digital Content, http://links.lww.com/SMJ/A230 and http://links.lww.com/SMJ/A231. A brief explanation of the OSI nomenclature is given on p. 1 of the Supplement herein. Part II contained queries about burnout using the CBI [27] and sleep using the PSQI [46]. We computed the scores for Personal (A), Work-related (B) and Patient-related Burnout (C) and for each of the seven components comprising the total PSQI. Each component of the questionnaires was coded blindly. A total of 109 physicians completed Part I. Altogether 76 physicians completed both parts, whereas 14 physicians only filled in part II. Available information about the non-participating and partially-participating physicians was presented by Goyal et al. [4].

### 2.4 Qualitative data: Open-ended questions

At the end of the OSI was a series of open-ended questions. The physicians were asked about the best and hardest part of their work. They were requested to suggest how the difficulties could be diminished or eliminated/how work conditions could be improved. Those who stated that they lacked genuine rest breaks were asked to indicate what the hindrance(s) were. There were other opportunities for open-ended replies, e.g. if pointless tasks were performed, the physician was asked to describe these. Responses to the open-ended queries were evaluated blindly with regard to the quantitative data. Qualitative content analysis [47, 48] of these open-ended answered was performed independently by each of the authors. Thereafter, the assessments were discussed until agreement was achieved.

### 2.5 Parallel mixed methods design ∼ Integration

A parallel mixed methods design was used, namely, the quantitative and qualitative data were collected at the same time with synthesis performed thereafter [42]. We specially targeted integrative analysis towards areas for potential intervention.

### 2.6 Statistical analysis

Initially, extensive univariate analyses were undertaken. Broadly consistent with Wasserman and Ossiander [49], to preserve confidentiality we avoid stating the precise number for five or fewer participants. Pearson and Spearman correlation, Pearson χ^2^ or Fisher’s exact test were used for bivariate analysis. Multicollinearity was assessed, with the upper limit of Pearson’s *r* = 0.70. We performed multivariable regression analysis [50] as follows. The relation between the independent variables (work stressors as assessed with the OSI questionnaire) and each of the burnout and sleep components, as well as the total PSQI, was evaluated via multiple linear regression with statistically significant results (*p* < 0.05) presented. Insofar as there was at least one statistically significant multivariable association between a job stressor and an outcome measure, borderline multivariable associations (generally 0.05 ≤ *p*≤0.06) between that job stressor and other outcome measures are also displayed. Multiple logistic regression was used to compute odds ratios (OR) and confidence intervals (CI) for dichotomous outcome variables. Statistical analysis was performed by means of Statistica software (14.0.0.15, 2021 TIBCO version).

## 3 Results

### 3.1 Information from the institute records

All physicians employed at the Institute were on salary, i.e. their pay was fixed. They were not permitted to take on any outside employment. Most of the physicians took night call, with the exception of those within the community medicine specialty area. All physicians worked full-time, at least 5 days per week.

### 3.2 Quantitative data analysis from the questionnaire

Table 1 provides a summary of salient demographic data, total OSI, the OSI levels and aspects, the three CBI scales, and the total PSQI and the PSQI components. Internal consistency, evaluated by the Cronbach α, for the total OSI revealed α= 0.80, for burnout (A) α= 0.88, for burnout (B) α= 0.89 and for burnout (C) α= 0.92. For the PSQI, the Cronbach α= 0.71. The extensive univariate findings from the OSI questionnaire are presented in the Supplement.

**Table 1:** Occupational Stressor Index, Burnout, Sleep and other salient univariate data for the participating physicians

	N	Mean	Sd	Median	IQR
Age	123	29.7	5.83	29	7
Working years as a physician (0 = < 1y, 1 = 1–5 y, 2 = 5–10y, 3 > 10y)	109	1.22	0.81	1	1
Total OSI	106	87.4	8.09	86.8	10.5
OSI level totals
Input	107	22.9	2.11	23.0	3.0
Central decision-making	109	18.5	1.10	18.5	2.0
Output/task performance	109	15.3	2.49	15.4	2.5
General	107	30.5	4.81	30.1	6.6
OSI aspect totals
Underload	108	3.9	1.15	4.0	1.5
High demand	107	31.2	2.87	32.0	3.4
Strictness	108	17.2	2.67	17.4	4.5
External time pressure	109	7.7	1.12	7.75	1.5
Noxious exposures	109	2.0	1.36	1.75	1.75
Threat avoidance/symbolic aversiveness	109	10.6	2.25	10.5	3.5
Conflict/uncertainty	108	14.7	2.37	14.5	3.0
Copenhagen Burnout Index
Personal Burnout (A)	90	45.9	17.9	45.8	25.0
Work-related Burnout (B)	90	40.5	20.2	44.6	28.6
Patient-related Burnout (C)	90	30.2	22.1	29.2	33.3
Global PSQI Score (Sum of the 7 components)	89	6.07	3.06	6.0	3.0
PSQI components:
1: Subjective sleep quality	90	1.08	0.64	1.0	0
2: Sleep latency	90	0.88	0.89	1.0	1.0
3: Sleep duration	89	1.71	0.71	2.0	1.0
4: Habitual sleep efficiency	89	0.36	0.76	0	0
5: Sleep disturbance	90	0.83	0.57	1.0	0
6: Sleep medication (prescribed or over the counter)	90	0.19	0.62	0	0
7: Daytime dysfunction	90	1.02	0.82	1.0	2.0
Gender		%
Male	84	68.3
Female	39	31.7
Level of training
Junior resident	54	49.5
Senior resident	35	32.1
Attending physician	20	18.4
Specialty area
Surgical/anesthesia/emergency medicine	52	47.7
Internal medicine/pediatrics/psychiatry/dermatology/	43	39.5
combined specialties &subspecialties
Community medicine	14	12.8

IQR = Interquartile range, PSQI = Pittsburgh Sleep Quality Index, OSI = Occupational Stressor Index, Sd = standard deviation, *y* = years.

Age and work years as a physician were closely correlated (Pearson *r* = 0.74); the latter co-variate was chosen for multivariable analysis. All multivariable analysis also includes gender as a co-variate. Table 2 shows that a significant OR is observed with unit change in the total OSI for burnout (A) and (B), as well as for the PSQI, but not for burnout (C). In Table 2, the outcome variables were dichotomized at the integer median cutpoint.

**Table 2:** Multivariable logistic regression analysis of the relation between the total Occupational Stressor Index and the Copenhagen Burnout Indices and the Pittsburgh Sleep Quality Index

	Personal Burnout (A) >46 *N* = 74			Work-related Burnout (B) >45 *N* = 74			Patient-related Burnout (C) >29 *N* = 74			PSQI ≥ 6 *N* = 73		
	OR	–95% CI	+95% CI	OR	–95% CI	+95% CI	OR	–95% CI	+95% CI	OR	–95% CI	+95% CI
Work years as a physician	0.91	0.48	1.73	0.99	0.51	1.91	0.93	0.49	1.79	0.36 (*p* = 0.008)	0.16	0.77
Gender (male)	1.93	0.62	6.00	3.18	0.97	10.5	5.39 (*p* = 0.004)	1.71	17.0	2.68	0.75	9.57
Total OSI (unit change)	1.10 (*p* = 0.01)	1.02	1.18	1.12 (*p* = 0.005)	1.04	1.22	1.02	0.96	1.10	1.09 (*p* = 0.03)	1.01	1.17
Model χ^2^	8.52 (*p* = 0.04)			12.5 (*p* = 0.006)			9.52 (*p* = 0.02)			16.2 (*p* = 0.001)		

CI = confidence interval, OR = odds ratio, OSI = Occupational Stressor Index, PSQI = Pittsburgh Sleep Quality Index.

The queries from the OSI questionnaire, as well as the OSI elements (whose acronyms are indicated) showing multivariable associations with burnout and/or the PSQI are presented in Table 3. This starts with the OSI questionnaire sub-section C related to Work Hours and Scheduling. Approximately 46% of physicians usually worked 7 days/week. Among the 76 physicians who also completed part II, an increased risk of work-related burnout was associated with working 7 days/week. Over half of the physicians had fewer than 2 weeks of paid vacation truly free from work. Lack of paid vacation truly free from work showed a multivariable association with personal and work-related burnout, as well as with daytime dysfunction (component 7 of the PSQI). Several characteristics of rest break insufficiency were linked to burnout and/or compromised sleep. The majority of physicians had infrequent rest breaks of short duration without being genuinely free from work obligations. All physicians reported working over two hours without any rest break whatsoever.

It is seen that the physicians without possibilities for upgrade, as well as those who lacked full support for career advancement, had diminished habitual sleep efficiency. Insufficient recognition of good work showed a significant multivariable relation to poorer sleep quality, as well as a borderline association with work-related burnout.

Several of the queries regarding working conditions, as per sub-section E, showed multivariable associations with burnout and/or sleep. These include exposure to glare (in the operating room or elsewhere) and patient-related burnout, to visually distressing scenes and sleep disturbances, and to radiation with personal burnout, as well as PSQI (borderline for the latter). As noted on p. 4 of the Supplement, very few of the physicians had their own office; most did not have their own work desk and nearly half considered their office to be cramped. Up to 11 physicians shared a single office. Several multivariable associations were associated with percent of time spent in the office; namely, with personal as well as work-related burnout and daytime dysfunction and borderline for PSQI. Working in an office without an adequate window was also associated with poorer subjective sleep quality.

Sub-section F of the OSI questionnaire addresses mishaps at work. The relatively few physicians who had heard about or witnessed a serious or fatal work accident had increased personal and patient-related burnout scores. Reports of suicide attempts or completed suicide among colleagues or staff at work showed multivariable associations with personal burnout, sleep disturbances and PSQI. Few of the physicians stated that they knew there was a properly-functioning system in place for non-medical emergencies. The lack of such was associated with poorer subjective sleep quality (*p* = 0.05) (not shown in Table 3) as well as shorter sleep duration (*p* = 0.006).

Sub-section G of the OSI questionnaire was concerned with time pressure. When manifested in conflict between workload and time constraints, multivariable associations were seen with all three types of burnout, as well as with daytime dysfunction.

**Table 3:** Queries from the OSI showing significant or near significant multivariable associations with

C. WORK HOURS &SCHEDULING														
	Personal Burnout (A) *N* = 76			Work-related Burnout (B) *N* = 76			Patient-related Burnout (C) *N* = 76			Most significant PSQI component			PSQI *N* = 75		
ß	B	SE of B	ß	B	SE of B	ß	B	SE of B	ß	B	SE of B	ß	B	SE of B
Usual # workdays/week				0.24 (*p* = 0.048)	8.89	4.43
									*Daytime Dysfunction N* = 76		
Lacks work-free paid vacation (GH6)	0.26 (*p* = 0.03)	6.21	2.80	0.27 (*p* = 0.02)	7.59	3.15				0.30 (*p* = 0.009)	0.32	0.12
									*Poorer Subjective Sleep quality N* = 76		
Infrequent rest breaks	0.22 (*p* = 0.06)	9.78	5.20	0.25 (*p* = 0.03)	12.8	5.83				0.32 (*p* = 0.005)	0.50	0.17	0.20 (*p* = 0.08)	1.53	0.85
Only short rest breaks										0.26 (*p* = 0.02)	0.72	0.30
									*Shorter Sleep Duration N* = 75		
Not true rest breaks										0.32 (*p* = 0.006)	1.03	0.36
D. SALARY, ADVANCEMENT POSSIBILITIES, RECOGNITION														
	**Personal Burnout** (A) *N* = 76			**Work-related Burnout** (B) *N* = 76			**Patient-related Burnout** (C)			**Most significant PSQI component**			**PSQI**		
	ß	B	SE of B	ß	B	SE of B	ß	B	SE of B	ß	B	SE of B	ß	B	SE of B
									*Poorer Habitual sleep efficiency N* = 75		
Lacks possibilities for upgrade										0.31 (*p* = 0.006)	0.29	0.10
										*N* = 62
Lacks support for upgrade										0.28 (*p* = 0.02)	0.36	0.16
									*Poorer Subjective Sleep quality N* = 76		
Lacks recognition of good work (GU4)				0.22 (*p* = 0.06)	9.49	5.01				0.25 (*p* = 0.03)	0.33	0.15
E. WORKING CONDITIONS														
	**Personal Burnout** (A) *N* = 76			**Work-related Burnout** (B) *N* = 76			**Patient-related Burnout** (C) *N* = 76			**Most significant PSQI component**			**PSQI** *N* = 75		
	ß	B	SE of B	ß	B	SE of B	ß	B	SE of B	ß	B	SE of B	ß	B	SE of B
Glare exposure (INOX1)							0.26 (*p* = 0.02)	10.1	4.19
									*Sleep disturbances N* = 76		
Visually disturbing scenes										0.25 (*p* = 0.03)	0.24	0.11
Radiation exposure	0.26 (*p* = 0.03)	4.76	2.13										0.22 (*p* = 0.05)	0.69	0.35
N = 75			N = 75						*Daytime Dysfunction N* = 75			N = 74		
Percent time in office	0.26 (*p* = 0.03)	0.15	0.07	0.28 (*p* = 0.02)	0.19	0.07				0.24 (*p* = 0.04)	0.006	0.003	0.22 (*p* = 0.055)	0.02	0.01
									*Poorer Subjective Sleep quality N* = 76		
Lacks needed window in office										0.24 (*p* = 0.03)	0.43	0.20
F. MISHAPS AT WOR K														
	**Personal Burnout** (A) *N* = 76			**Work-related Burnout** (B) *N* = 76			**Patient-related Burnout** (C) *N* = 76			**Most significant PSQI component**			**PSQI**		
	ß	B	SE of B	ß	B	SE of B	ß	B	SE of B	ß	B	SE of B	ß	B	SE of B
Injury to colleagues/staff	0.27 (*p* = 0.02)	13.5	5.69				0.33 (*p* = 0.002)	20.5	6.4
	*N* = 75									*Sleep disturbances N* = 75			*N* = 74		
# suicide attempts/ completed suicides at work	0.23 (*p* = 0.05)	4.62	2.36							0.233 (*p* = 0.045)	0.15	0.07	0.31 (*p* = 0.007)	1.05	0.38
	*N* = 76												N = 75		
Suicide attempt or completed suicide of known person at work	0.24 (*p* = 0.04)	10.4	5.04										0.23 (*p* = 0.047)	1.70	0.84
									*Shorter sleep duration*		
Lacking functioning system for non-medical emergencies (GAVOI5)										0.31 (*p* = 0.006)	0.37	0.13
G. TIME PRESSURE														
	**Personal Burnout** (A) *N* = 76			**Work-related Burnout** (B) *N* = 76			**Patient-related Burnout** (C) *N* = 76			*Daytime Dysfunction N* = 76			**PSQI**		
	ß	B	SE of B	ß	B	SE of B	ß	B	SE of B	ß	B	SE of B	ß	B	SE of B
Workload vs time constraint conflict	0.38 (*p* = 0.0008)	18.7	5.37	0.42 (*p* = 0.0002)	23.4	5.95	0.32 (*p* = 0.003)	19.2	6.28	0.25 (*p* = 0.03)	0.54	0.24
H.PROBLEMS/RESTRICTIONS/CONSTAINTS &INFLUENCE AT WORK														
	**Personal Burnout** (A) *N* = 76			**Work-related Burnout** (B) *N* = 76			**Patient-related Burnout** (C) *N* = 76			*Daytime Dysfunction N* = 76			**PSQI**		
	ß	B	SE of B	ß	B	SE of B	ß	B	SE of B	ß	B	SE of B	ß	B	SE of B
Lacks influence on # patients / scheduling										0.25 (*p* = 0.03)	0.27	0.13
Lacks influence over clinical tasks										0.26 (*p* = 0.03)	0.31	0.14
Interruptions from people hamper task performance (OCNFL3)	0.35 (*p* = 0.003)	11.8	3.78	0.43 (*p* = 0002)	16.4	4.12				0.302 (*p* = 0.008)	0.45	0.16
I. INTERPERSONAL INTERACTIONS &SOCIAL CLIMATE														
	**Personal Burnout** (A) *N* = 76			**Work-related Burnout** (B) *N* = 76			**Patient-related Burnout** (C) *N* = 76			*Longer sleep latency N* = 76			**PSQI** *N* = 75		
	ß	B	SE of B	ß	B	SE of B	ß	B	SE of B	ß	B	SE of B	ß	B	SE of B
Abuse of power/violations of behavior norms (GCNFL4)	0.31 (*p* = 0.01)	13.1	5.05	0.43 (*p* = 0.0002)	21.0	5.43	0.24 (*p* = 0.04)	12.5	5.90	0.34 (*p* = 0.004)	0.73	0.25
									*Poorer Subjective Sleep quality*		
Lacks grievance redress (GCNFL5)	0.23 (*p* = 0.046)	6.51	3.20	0.20 (*p* = 0.08)	6.38	3.64				0.22 (*p* = 0.05)	0.21	0.11	0.30 (*p* = 0.007)	1.47	0.53
J. WORKLOAD &ACTIVITIES														
	**Personal Burnout** (A) *N* = 76			**Work-related Burnout** (B) *N* = 76			**Patient-related Burnout** (C) *N* = 76			*Daytime Dysfunction N* = 76			**PSQI** *N* = 75		
	ß	B	SE of B	ß	B	SE of B	ß	B	SE of B	ß	B	SE of B	ß	B	SE of B
Handles patients who can’t give a history	2.44 (*p* = 0.036)	6.82	3.19	0.255(*p* = 0.027)	8.12	3.59	0.254 (*p* = 0.019)	8.72	3.64	0.262 (*p* = 0.019)	0.32	0.14	0.245 (*p* = 0.029)	1.18	0.53
									*Poorer habitual sleep efficiency N* = 74		
Number of patients in ICU										0.293 (*p* = 0.01)	0.23	0.09
Several people seek attention simultaneously	0.27 (*p* = 0.02)	7.22	3.06	0.25 (*p* = 0.03)	7.58	3.48
									*Daytime Dysfunction N* = 76		
No separate time for non-clinical duties	0.28 (*p* = 0.015)	12.3	4.90	0.24 (*p* = 0.036)	12.0	5.60				0.214 (*p* = 0.056)	0.41	0.21
Under pressure to publish	0.28 (*p* = 0.02)	24.8	10.1	0.37 (*p* = 0.001)	37.3	11.0	0.33 (*p* = 0.002)	36.2	11.3	0.23 (*p* = 0.039)	0.92	0.44
									*Poorer Subjective Sleep quality N* = 76		
Performs invasive procedures							0.23(*p* = 0.037)	12.2	5.72	0.30 (*p* = 0.009)	0.44	0.17	0.24 (*p* = 0.04)	1.69	0.81
									*Longer sleep latency N* = 76		
Performs work of other personnel	0.27 (*p* = 0.02)	10.3	4.32	0.28 (*p* = 0.02)	11.9	4.87				0.24 (*p* = 0.04)	0.45	0.22	0.22 (*p* = 0.05)	1.41	0.71
									*Daytime Dysfunction N* = 76		
Performs pointless tasks (GCNFL7)	0.22(*p* = 0.057)	9.89	5.11	0.28 (*p* = 0.02)	14.0	5.69				0.41 (*p* = 0.0002)	0.80	0.20	0.26 (*p* = 0.02)	1.97	0.82
K. RECENT CHANGES AT WORK														
	**Personal Burnout** (A) *N* = 76			**Work-related Burnout** (B) *N* = 76			**Patient-related Burnout** (C) *N* = 76			*Daytime Dysfunction N* = 76			**PSQI**		
	ß	B	SE of B	ß	B	SE of B	ß	B	SE of B	ß	B	SE of B	ß	B	SE of B
↑ time pressure/deadlines	0.28 (*p* = 0.02)	10.4	4.20	0.38 (*p* = 0.001)	15.9	4.57	0.19 (*p* = 0.09)	8.51	4.93	0.24 (*p* = 0.04)	0.38	0.18

Multiple linear regression analysis with working years as a physician and gender included as covariates throughout. GAVOI = Threat avoidance on the general level. GH = high demand on the general level, GU = Underload on the general level, IAVOI = Threat avoidance on the input level, ICU = intensive care unit, INOX = noxious physical exposures on the input level, GCNFL = Conflict/uncertainty on the general level, GEP = Extrinsic time pressure on the general level, GS = Strictness on the general level, OCNFL = Conflict/uncertainty on the output level, OSI = Occupational Stressor Index, PSQI = Pittsburgh Sleep Quality Index, SE = standard error.

Problems, restrictions and influence at work were the focus of sub-section H. Lack of influence over the number of patients and patient scheduling, as well as over clinical tasks, were associated with daytime dysfunction. Longer sleep latency was also associated with lack of influence over clinical tasks (*p* = 0.04) (not shown in Table 3). An element of conflict on the output level within the OSI is interruptions from people hampering task performance. These interruptions showed powerful multivariable associations with personal and work-related burnout as well as with daytime dysfunction.

Sub-section I of the OSI questionnaire addresses interpersonal interactions and social climate. Abuse of power/violations of norms of behavior, an element of conflict on the general level, was reportedly very unusual among the participating physicians. However, even when rare, this was significantly associated with all three types of burnout and longer sleep latency, as well as daytime dysfunction (*p* = 0.04) (the latter not shown in Table 3). Only about half the participants stated that there was an efficient, confidential system in place for redressing grievances, also an element of conflict on the general level. Lack of such a grievance procedure showed a multivariable association with personal burnout and PSQI, and borderline for work-related burnout and poorer subjective sleep quality.

Workload and activities are the focus of sub-section J. Therein, handling patients who cannot give a history was related to all 3 types of burnout, daytime dysfunction and PSQI. Over half of the physicians were responsible for patients in intensive care. The larger the number of such patients, the poorer was habitual sleep efficiency. Having several people seeking one’s attention simultaneously was a frequent occurrence, associated with personal and work-related burnout. As could be anticipated in the academic medical setting, all physicians had other duties besides clinical work. Most had no separate time allocated for these other duties; the lack of which was associated with personal and work-related burnout, and borderline significantly associated with daytime dysfunction. Over half of the physicians performed these non-clinical tasks outside work hours, associated with poorer sleep quality (*p* = 0.02) and daytime dysfunction (*p* = 0.07) (results not shown in Table 3). The majority of physicians indicated that they were under some or heavy pressure to publish/present new findings at congresses and meetings outside their institution. Such pressure was associated with all three types of burnout, as well as daytime dysfunction. The vast majority of the physicians performed invasive procedures. This was associated with patient-related burnout and poorer sleep quality, as well as PSQI. Nearly 30% of the physicians reported performing work of other personnel, associated with personal and work-related burnout, as well as longer sleep latency, and borderline with PSQI. Performing tasks that seem pointless also showed a relation to work-related burnout, as well as with daytime dysfunction and PSQI, and borderline for personal burnout. Section K addresses recent changes at work. Over 40% of the participating physicians reported an increase in time pressure/number of deadlines. Those who responded affirmatively to that query showed increased personal and work-related burnout as well as daytime dysfunction. A borderline association was observed for patient-related burnout.

### 3.3 Qualitative data/content analysis

#### 3.3.1 Witnessing or hearing about injury to colleagues/staff

Among the 26 physicians who had either witnessed or heard about serious or fatal injury to colleagues or staff, 11 described what had happened. The majority of the injuries were due to an assault from a patient, the patient’s family or an attender, i.e. physical violence. There were also reported needle sticks and a few other accidents.

#### 3.3.2 Abuse of power/violations of norms of behavior

As noted, abuse of power/violations of norms of behavior were reported by a very small number of the physicians. Among these, a few were described. These include being stopped from taking academic leave and other forms of forced duty, as well as physicians in more advanced levels mistreating junior residents.

#### 3.3.3 Pointless tasks and/or tasks of other personnel

**Fig. 1:**
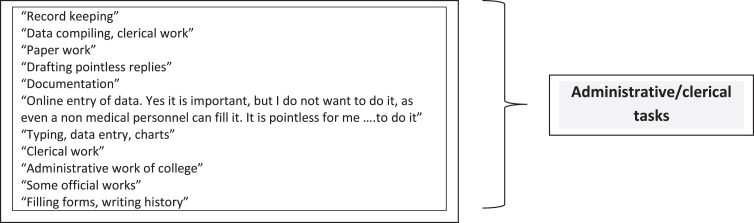
Performing pointless tasks &/or tasks of other personnel.

Among those who stated that they performed pointless tasks and/or tasks of other personnel, altogether 11 physicians described these tasks. The initial responses are on the left side of Fig. 1. These all appear to be related to administrative/clerical work, as classified on the right side of the figure.

#### 3.3.4 The reasons why genuine rest breaks are lacking

**Fig. 2:**
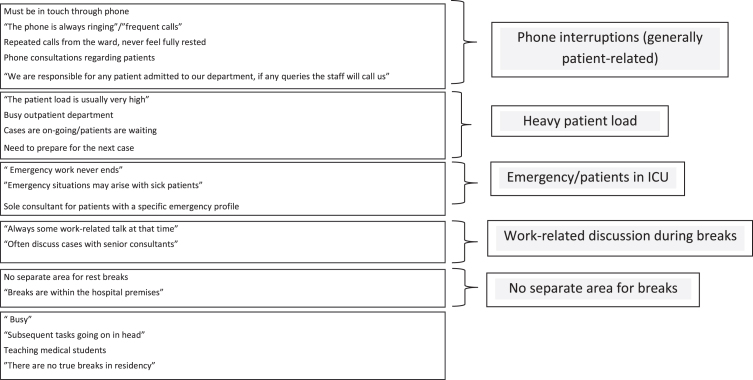
Why genuine rest breaks are lacking.

As noted, the vast majority 82 (75.2%) of the participating physicians stated that they did not have true rest breaks free from work obligations. At least one reason for lacking genuine breaks was specified by 39 (47.6%). The most frequently given response was related to phone interruptions that were mainly patient-related, cited by thirteen of the physicians (Fig. 2). Heavy patient load and the special demands of emergency work were also often listed. Other reasons for lacking genuine rest breaks work were attributed to having work-related discussions during breaks, as well as lack of separate rest areas.

#### 3.3.5 Most difficult part of work as a physician in the respective profile

**Fig. 3:**
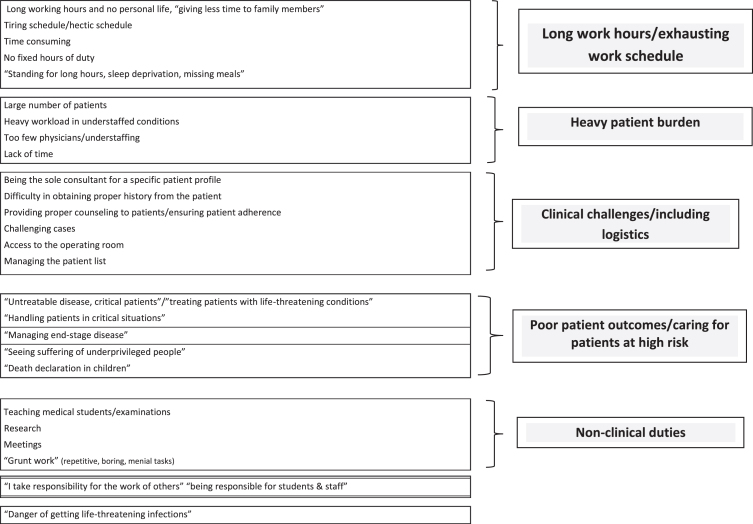
Most difficult part of work as a physician in one’s respective profile.

A total of 70 of the 109 physicians (64.2%) responded to the open-ended query regarding the hardest part of work in their respective profile. The most frequently cited difficulty was long work hours/exhausting work schedule, noted by 17 of the participants. The other often mentioned difficulties can be categorized as heavy patient burden, clinical challenges, poor patient outcome/caring for patients at high risk, as well as performance of non-clinical duties, as displayed in Fig. 3.

#### 3.3.6 Best part of work as a physician in the respective profile

**Fig. 4:**
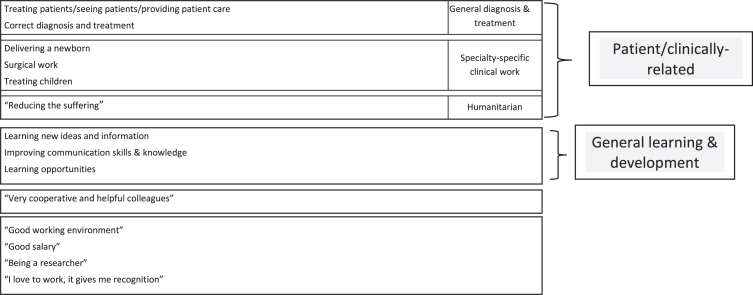
Best part of work as a physician in one’s respective profile.

Altogether 25 of the physicians answered the open-ended question regarding the best part of work in one’s respective profile. This was fewer than those physicians who responded to the query about the hardest part of their work (Pearson χ^2^ = 37.8, *p* < 0.0001). The 82 physicians who did not respond to this query had a higher total mean high demand score: 31.5 ± 2.4 compared to the 25 who specified the best part of their work 29.9 ± 4.0 (*t* = 2.49, *p* = 0.01). Among the 25 physicians who gave an answer to the query, 13 (52%) named patient/clinically-related aspects as the best part of their work, followed by general learning and development, as presented in Fig. 4.

#### 3.3.7 Suggestions to improve work/ameliorate burdens

**Fig. 5:**
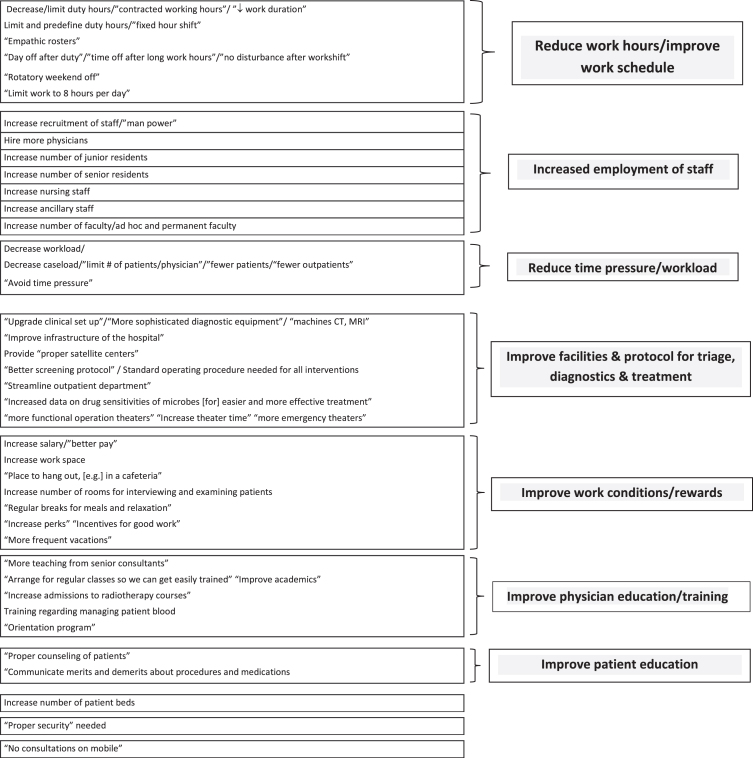
Suggestions to improve work/ameliorate burdens.

Ninety of the 109 physicians (82.5%) made at least one suggestion as to how their work could be improved and/or how the difficulty could be ameliorated. These are shown in Fig. 5. Among the most frequently recommended was to reduce work hours/improve work schedule, suggested by 22 of the physicians. Increased employment of staff was even more often recommended, altogether by 35 of the physicians. In many instances, this suggestion was made in conjunction with the need to reduce work hours. Other suggestions included improvement in facilities, triage, diagnostic and treatment, work conditions/rewards, physician education and training and patient education.

## 4 Discussion

### 4.1 Consolidation of the qualitative and quantitative analyses aimed at informing interventions

Further insights, especially those of potential relevance for interventions, can be garnered from an integrative assessment of the quantitative and qualitative findings, including some further salient bivariate and multivariable analysis, as will now be explored.

#### 4.1.1 Work hazards/potentially disastrous occurrences

There was a multivariable association between having witnessed or heard about a serious or fatal accident to colleagues and/or staff and personal as well as patient-related burnout. When described, these incidents were mainly associated with physical violence from a patient, the patient’s family or an attender. Over 25% of the physicians reported threat of violence as an acute work hazard. Notably, only about 25% of the physicians indicated that there was a system in place for non-medical emergencies and that they were certain of its proper functioning. The present analysis, moreover, suggests that sleep duration is shortened by the lack of such a system. It can therefore be recommended that such a system be fully operative, that all physicians know this and have ready access to it whenever needed, especially as a protection against assault.

Altogether 89 (82%) of the physicians answered that they faced risk of infection as a work hazard. This was just before the onset of the COVID-19 pandemic, as noted. These issues obviously became even more vital subsequently. A recent study of dentists in Andhra Pradesh, India, indicated that provision of dental care was associated with an OR of 2.8 (95% CI 2.4 –3.4) for COVID19 infection. The need was underscored for proper implementation of safe practices [51]. In this light, attention is also warranted to the physical facilities, i.e. work offices, described as cramped by nearly half of the physicians, with a mean of five people sharing a single office, and few of the physicians having their own work desk. The impact of these adverse office conditions is suggested by the multivariable association between percent of time spent in the office with personal and work-related burnout, as well as with daytime dysfunction.

Another work hazard is radiation exposure, reported by over 40% of the physicians, and associated with personal burnout. Among those who reported exposure, only about one-third stated that they wore a radiation badge. Remedying this situation could have a multi-faceted impact, to monitor exposure, per se, as well as being a reflection of concern for the physicians’ health and safety.

Suicide attempts or completed suicide among colleagues and staff, particularly insofar the person was known to the physician, were also found to impact upon personal burnout as well as sleep disturbances.

The general level of the threat avoidance/symbolic aversiveness aspect of the OSI considers mishaps at work, several of which were found to impact burnout and/or sleep. Namely, having witnessed or heard about injury to colleagues and/or staff (GAVOI2) and suicide at work (GAVOI4). Together with lack of a functioning emergency system (GAVOI5), having experienced injury at work (GAVOI1) as well as litigation/testifying in court regarding work (GAVOI3), the sum of these five OSI elements yields the total general level threat avoidance/symbolic aversiveness score (GAVOIT). As seen in Table 4, there is a multi-variate association between GAVOIT and personal burnout.

**Table 4:** The relation between total general level threat avoidant vigilance/symbolic\\ aversiveness and personal burnout

	Personal Burnout (A) *N* = 76		
	ß	B	SE of B
Working years as MD	–0.03	–0.62	2.67
Gender (male)	–0.04	–1.63	4.56
General level threat avoidance/symbolic aversiveness (GAVOIT)	0.248 (*p* = 0.03)	2.91	1.37
R^2^ (Adjusted R^2^)	0.07 (0.03)		
Model F	1.70 (*p* = 0.17)		

Multiple linear regression analysis. GAVOIT = total general level threat avoidance vigilance, SE = standard error.

When viewed in concert, these findings emphasize the need to take past and present aversive work-related experiences into full consideration. Support and help should be provided to prevent adverse outcomes. Concordantly, the deleterious impact of threat avoidant vigilance/symbolic aversiveness among physicians vis-à-vis other health outcomes/lifestyle-related risk factors (hypertension, smoking, obesity) has been reported [20, 22]. As emphasized by Chiavarino et al. [52], confidence of health professionals to identify and manage hazards should be bolstered.

#### 4.1.2 Abuse of power/violations of norms of behavior

Albeit unusual in this cohort, reported abuse of power/violations of the norms of behavior showed multivariable associations with all three types of burnout, as well as with longer sleep latency and daytime dysfunction. These violations are akin to the construct termed relational or organizational injustice, prospectively associated with burnout [53], a consideration that has become even more salient for health care providers during the COVID-19 pandemic [54].

The few descriptive responses suggested that forced duty was one of the manifestations. In support of that qualitative finding, the 43 physicians who reported working during paid vacation had a higher mean score on abuse of power/violations of the norms of behavior (0.36 ± 0.61) compared to the 66 physicians who did not report working during paid vacation (0.05 ± 0.14) (*t* = 4.0, *p* = 0.0001).

There was also a correlation between the scores for abuse of power/violations of the norms of behavior and insufficient recognition of good work (Spearman *ρ*= 0.32, *p* = 0.0006). Recall that the latter variable showed multivariable associations with work-related burnout and poor subjective sleep quality. An association was found, as well, between abuse of power/violations of the norms and tension with display of knowledge (Spearman *ρ*= 0.21, *p* = 0.03). Yet another deleterious trajectory is seen in further analysis. Namely, interruptions from people hampering task performance were correlated with abuse of power/violations of the norms of behavior (Spearman *ρ*= 0.20, *p* = 0.035). As noted, these interruptions themselves, were linked to personal and work-related burnout, as well as to daytime dysfunction. Avoiding unnecessary interruptions should be a basic norm of behavior, reflecting respect for physicians, as well as for others. When such a climate of respect is lacking, a platform is warranted to confidentially report and effectively address issues of concern. For the present cohort, upgrading such a platform appears to be needed. Notably, only about 40% of the physicians stated that redress of grievances could be done in an efficient and confidential manner.

We now segue to another association regarding abuse of power and violations of norms of behavior. Namely, the 26 physicians who reported performing pointless tasks had a higher mean score on abuse of power/violations of the norms of behavior (0.37 ± 0.64) compared to the 83 physicians who did not report performing pointless tasks (0.11 ± 0.31) (*t* = 2.8, *p* = 0.007). This finding coheres with the recognized relation between performing unnecessary or unreasonable tasks and unfairness at work [55].

#### 4.1.3 Performance of tasks that seem pointless

Pointless task performance was reported by nearly 25% of the physicians, showing multivariable association with personal and work-related burnout, daytime dysfunction and total PSQI. Concordantly, among Danish human-service workers performing unnecessary work tasks predicted subsequent mental-health deterioration [56]. The importance of eliminating unnecessary tasks has been particularly underscored for the well-being of physicians and other health care employees [57, 58].

Our qualitative analysis suggests that all these tasks were of an administrative/clerical nature. Moreover, a strong association was found between performing tasks of other personnel and performing pointless tasks (Pearson χ^2^ = 7.02, *p* = 0.008). It is deemed feasible for this Academic Medical Institute to continually evaluate how much record keeping is truly essential, and, also, to train and assign non-clinical staff to these activities. The potential impact of this intervention can be inferred from a study indicating that training clerical support staff to enter doctors’ orders reduced burnout and improved work satisfaction among physicians [59].

Further indication for the importance of this intervention is suggested by associations between performing pointless tasks and other burdens faced by these physicians. Namely, working during paid vacation and performing pointless tasks yielded a Pearson χ^2^ of 6.98 (*p* = 0.008). In addition, pointless tasks appear to compromise the possibility of having genuine rest breaks, as indicated by a Pearson χ^2^ of 5.32 (*p* = 0.02) between these two variables.

#### 4.1.4 Insufficient genuine rest breaks

The impact of insufficient genuine rest breaks was indicated in several multivariable associations with compromised sleep as well as with burnout. Concordant findings were reported among emergency health professionals working in a French university hospital, namely a linkage between lacking adequate time or even completely skipping meals and burnout [60]. The reasons for this lack were multifold, yet predominantly patient-related. On the one hand, interventions on the individual level are necessary to encourage the physicians to take true breaks whenever possible, including turning off their phones and engaging in relaxing/refreshing endeavors. Culturally-coherent relaxation training could be helpful in this regard. Organizational and broader measures are indispensable for this to become a reality. Namely, rest breaks should be considered a vital part of the physician’s work routine. Double coverage/a ‘float’ physician would be needed to take phone calls and otherwise cover patient-related issues, so that the physician is genuinely free from work for shorter and longer periods throughout his/her time on-duty. An easily accessible, quiet, dedicated rest area would be a key concomitant. These considerations are particularly salient for physicians who handle patients in emergency status.

As seen in Table 5, handling a larger percentage of patients in emergency status was associated with a greater likelihood of lacking genuine rest breaks. In light of the setting within an Academic Medical Institute, in which all 109 participating physicians had other duties besides clinical work, it is also noteworthy that those physicians who did not have separate time for non-clinical duties were significantly less likely to have true rest breaks. This finding underscores the need to allocate work time for non-clinical duties, and to thereby ensure that rest breaks are not compromised. Adequate, genuine rest breaks would be among the most feasible, immediate interventions. It has been demonstrated that rest breaks do not impact adversely upon productivity [61] and if coupled with health-promoting practice can contribute to a paradigm shift for workplace culture [62].

**Table 5:** Factors significantly associated with lack of true rest breaks

No true rest breaks *N* = 109		
	OR	–95% CI	+95% CI
Work years as a physician	1.84	0.94	3.58
Gender (male)	0.30 (*p* = 0.03)	0.10	0.93
**Percentage of patients in emergency status**	2.20 (*p* = 0.038)	1.04	4.69
Model χ^2^		11.3 (*p* = 0.01)
Work years as a physician	1.65	0.85	3.21
Gender (male)	0.33 (*p* = 0.054)	0.10	1.03
**No separate time for non-clinical duties**	4.06 (*p* = 0.005)	1.52	10.8
Model χ^2^		14.5 (*p* = 0.002)

Multiple logistic regression analysis CI = confidence interval, OR = odds ratio.

#### 4.1.5 Long work hours/exhausting work schedule

There is substantial concordance between the quantitative and qualitative findings vis-à-vis long work hours/exhausting work schedule. The overall conclusion is that this is the most difficult part of the work for these physicians, with reduction in work hours and improving the work schedule among the most frequent recommendations. The clearest quantitative measure was working seven days per week, reported by nearly half of the participants, and associated with increased work-related burnout. The recommendation would therefore be to ensure that each physician has, at the very least, one off-duty day per week. The importance of guaranteeing complete off-duty days was underscored in a study of firefighters [63], wherein working during days that should have been off-duty adversely impacted sleep, as assessed by the PSQI. Notably, the work of firefighters and health professionals shares the common feature of heavy threat avoidant vigilance burden, whose deleterious impact requires particular attention, especially to ensuring adequate recovery time [14, 64].

#### 4.1.6 Most often recommended modification: Employment of more staff

A good number of the stressors found to be associated with burnout as well as compromised sleep, could be ameliorated insofar as more staff, especially physicians at various levels of training as well as nurses, were employed. With adequate staffing, it would be feasible to assign a ‘float’ physician, thereby providing coverage not only to ensure rest breaks, but also to avoid interruptions during the work shift, as well as to guarantee at least one day per week as completely free from work. With more clinical staff, separate work time could be allocated to non-clinical obligations, so that vacation time would be truly free from work. The need for truly work-free vacation is underscored by the multivariable associations between lack of such with personal and work-related burnout, as well as with daytime dysfunction.

### 4.2 Limitations

Since the independent variables and outcomes were self-reported using ordinate scales in the quantitative analysis, common method bias is a limitation of the present study [65]. This problem may have been somewhat mitigated since parts I and II were completed at different occasions. Further attenuation of common method bias may have been achieved by the consolidation of the qualitative and quantitative analysis.

The present investigation also has the limitation of a low participation rate, common to many studies of physician burnout, (e.g. [58, 66, 67]). This problem was exacerbated by the requirement that the questionnaires be completed when off-duty with no compensation for the time expended. Notably, the long work hour score was maximum for all 109 physicians who completed the OSI, such that off-duty time was exceedingly limited. In the study by Nedić et al. [23] the physicians also completed the OSI when off-duty and without compensation. However, the mean long work hour score was lower (1.65 ± 0.39) and full participation was achieved, albeit for a smaller number of physicians working in a single outpatient setting. Notably, in the present study, the physicians who completed only part I had more emergency cases (2-sample *t* test, *p* = 0.03), compared to those who completed both parts.

There was insufficient variance for night shift work in the present study. This limits the possibility to identify potential interventions in regard to that important stressor.

Residual (uncontrolled) confounding is also possible by other individual factors, such as marital status, number of children, comorbidities. Power limitations of the present study preclude stratified analysis of gender, as a key effect modifier in the relations between job stressors and health outcomes [68].

Due to the cross-sectional design of the present study, inferences cannot be made about temporal nature of the identified associations. The findings can, however, be helpful in informing future investigations, particularly of an interventional design. As noted, “observational research can inform decisions about which interventions might be most effective” [69] (p.144).

Some time has elapsed since the data were collected. In the meantime, the COVID-19 pandemic has further exacerbated the situation for these physicians. The added burden of the pandemic has made burnout among physicians and other health professionals including in the academic setting an even more pressing problem [70–74].

## 5 Conclusions

The present study offers a methodologically rigorous, in-depth approach, based upon the physician-specific OSI. The mixed methods design provides further insights. The integration can help inform the development of evidence-based interventions. Specific working conditions that may contribute to burnout and compromised sleep are identified among physicians working in the highly challenging setting of academic medicine. Thereby, the clarion call cited at the beginning of this paper [1] has been addressed, providing guidance as to how the mental health and work capacity of physicians might be better preserved and protected.

## Ethics statement

The Ethics Committee of the All-India Institute of Medical Sciences, Jodhpur approved the study (AIIMS/IEC/2018/564-May, 2018). Informed consent was requested from all participants, with full confidentiality guaranteed. The voluntary nature of participation was emphasized (no monetary incentives) with the freedom to withdraw without any consequences whatsoever. The study was performed in accordance with the Declaration of Helsinki of 1964 and its later amendments or comparable ethical standards.

## Conflict of interests

The authors declare that they have no conflict of interest.

## Supplementary Material

Supplementary Material
