# Supplementary material for: Job stressors in relation to burnout and compromised sleep among academic physicians in India
Source: Work. 2024 Jun 7;78(2):505–25. doi: 10.3233/WOR-230383 (PMC11191458; doi:10.3233/WOR-230383)
Supplement: Supplementary Material [file wor-78-wor230383-s001.pdf]

Univariate Data from the Physician-specific Occupational Stressor Index (OSI) Questionnaire

Further data about the work environment of the physicians, in addition to those presented in the main text, are provided in this Supplement. This generally follows the order of the physician-specific OSI questionnaire.

The physician-specific OSI questionnaire and score sheet are found at: Supplemental Digital Content, http://links.lww.com/SMJ/A230 and http://links.lww.com/SMJ/A231, respectively.

The main headings of the OSI questionnaire help orient the respondent. These are bolded and highlighted herein and labeled with a capital letter. All the questions within a given main heading are identified by that capital letter, followed by the number indicating the order of appearance.

For example: within the main heading **C**. **WORK HOURS & SCHEDULING**, question C7: Do you have the opportunity to take breaks during your workday?

One or more queries from the OSI questionnaire may be used to generate an OSI element, from which the actual scoring of the OSI is done. The OSI element is denoted by its acronym and number followed by the descriptive name in bolded capital letters.

Example: From the OSI questionnaire there are 5 quantitative questions regarding rest breaks: C7 Rest break frequency C8 Rest break length, C9 True rest breaks free from work obligations and C10 Length of time without even a short break. The answers to these 5 queries are used to generate the score for the OSI element: GH4 **LACK OF REST BREAKS**. Here, GH4, denotes the general level (G), high demand aspect (H) and 4 as the 4th OSI element within GH. In addition, there is an open-ended query within C9: If not true rest breaks, why not?

The OSI is organized as a 2-dimensional matrix. The top 3 rows correspond to information-transmission levels: input, decision making, and task performance. These are fundamental to cognitive ergonomics, facilitating objective analysis of stressors and their impact on the central nervous system [4]. The 4th level is general. The columns include 7 aspects (underload, high demand, strictness, extrinsic time pressure, noxious exposures, threat avoidant vigilance, and conflict/uncertainty). Each element of the OSI is scored from 0 (not present) to 2 (strongly present). The total OSI is the sum of all the scores of all the OSI elements. Further details about the numerical and theoretical basis of the OSI are found in Ref. [14]. A list of acronyms is provided on p. 9 of this Supplement.

As indicated in the main text, to maintain confidentiality, insofar as any result pertained to five or fewer of the physicians, we have avoided stating the exact number. Confidentiality is especially vital in this Supplement wherein univariate data are presented.

With this Supplement we aim to provide further insights into the work conditions of the participating physicians. Such data may be useful in meta-analyses.

==

A. LENGTH AND LOCATION OF WORK

==

A1, A3: **All 109 participants were full time employed as physicians**.

A2: **Years in current job at present site**

	Count	% Valid
< 6 months	46	42.2
≥ 6 months	63	57.8

A4: **Working years as a physician**

	Count	% Valid
< 1 year	15	13.8
1 to 5 years	66	60.6
6 to 10 years	17	15.6
> 10 years	11	10.1

Nearly all the participants had been employed only as physicians

======================================

B. TYPE OF PRACTICE/SETTING

======================================

B1, B2: **All 109 participants were employed at an Academic Teaching Hospital**

The levels of training and specialty areas of the participants are specified in Table 1 of the main text.

B3. **Percentage of inpatients under one's care**

	Code	Count	% Valid
< 10%	0	31	28.4
10% ≤ x ≤ 50	1	44	40.4
> 50%	2	34	31.2

B4. **Percentage of patients with end stage/incurable disease under one's care**

	Code	Count	% Valid
< 10%	0	59	54.1
10% ≤ x ≤ 20	1	31	28.4
20% < x < 50	1.5	10	9.2
≥ 50%	2	9	8.3

B5. **Percentage of patients in emergency status under one's care**

	Code	Count	% Valid
< 10%	0	51	46.8
10% ≤ x < 50	1	44	40.4
≥ 50%	2	14	12.8

B6. **All 109 physicians worked only at the one institution which was their sole employment.**

====================================

C. WORK HOURS & SCHEDULING

====================================

C1: **Length of usual work hours/day**

N	Mean	Standard deviation	Median	Interquartile range
109	13.8	3.0	12.0	4.0

C2: **Usual number of workdays/week**

N	Mean	Standard deviation	Median	Interquartile range
109	6.4	0.6	6.0	1.0

C4: **Calls/email outside work hours:** (0=never, 1=rarely, 2=occasionally, 3=frequently)

N	Mean	Standard deviation	Median	Interquartile range
109	1.76	1.0	2.0	2.0

C18 : **Works at home for job:** 0=never, 0.5=rarely, 1.5=sometimes, 2=frequently

N	Mean	Standard deviation	Median	Interquartile range
109	1.03	0.71	1.5	1.0

GH2 **LONG WORK HOURS**

0 Not more than 40h/week
0.5 40 to 42h/week
1 Frequently > 42h/week
2 Frequently > 48h/week

Calculate from C1-C2, add 0.5 points if C4=c or d (called or emailed at home)
or C18=c or d (works at home) and hours not included, to maximum 2 points,

N	Mean	Standard deviation	Median	Interquartile range
109	2	0	2	0

C5:GH6 **INSUFFICIENT PAID VACATION**

0 Over four weeks
1 Three to four weeks
1.5 Two weeks
2 Less than two weeks

Add 0.5 to 1 point if vacation is actually formal; work is performed during vacation to maximum 2 points.

N	Mean	Standard deviation	Median	Interquartile range
109	1.35	0.74	2.0	1.0

C7: **Rest break frequency**

2=Never 1.5=Rarely 1=Occasionally 0=Frequently

N	Mean	Standard Deviation	Median	Interquartile range
109	1.22	0.41	1.0	0.5

C8: **Rest break length**	N	%
Only short (< 15 minutes)	73	67.0
At least some breaks > 30 minutes	36	33.0

C9: **True rest breaks free from work obligations**	N	%
No	82	75.2
Yes	27	24.8

C10: **Over 2 hours without rest break**	N	%
Yes	109	100
No	0	

GH4 **LACK OF REST BREAKS**

0 C7 = frequently has rest breaks
1 C7 = occasionally has rest breaks
1.5 C7 = rarely has rest breaks
2 C7 =never has rest breaks

Add 0.5 points if C8=only short breaks, C9=not true rest breaks, if C10 > 2 hours, to a maximum of 2 points.

N	Mean	Standard Deviation	Median	Interquartile range
109	1.78	0.37	2.0	0.38

GH5: **NIGHT SHIFT WORK**

0 C3=regular work schedule AND C11=no night shift work
0.5 C3=irregular work schedule AND C11=no night shift work
1 C11=yes, night shift only
1.5 C11=yes, b (rotating night shift, up to four night shifts/month)
1.75 C11=yes, b (rotating night shift, at least once per week but not more than every fourth night)
2 C11=yes, b (rotating night shift, at least every third night)

Add 0.5 points if < 24 free time after working a rotating night shift, no guaranteed relief, up to maximum 2 points.

N	Mean	Standard Deviation	Median	Interquartile range
109	1.42	0.82	2.0	1.0

C16=GS4 **DIFFICULT TO TAKE TIME OFF**

0) Not at all 1.5) Somewhat
1) A little 2) Very much

N	Mean	Standard Deviation	Median	Interquartile range
109	1.35	0.50	1.5	0.5

C17= GS5 **WORK SCHEDULE INFLUENCE**

0) Complete, it is entirely up to me.
0.5) To a large extent, I mainly decide on my work schedule.
1.5) A little, but mainly my schedule depends on others, or is decided by others.
2) None at all, my schedule depends on others or is decided by others, and I have no say about it.

N	Mean	Standard Deviation	Median	Interquartile range
109	1.44	0.48	1.5	0

==

D. SALARY, POSSIBILITIES FOR ADVANCEMENT & RECOGNITION

==

D1 = GU1 **FIXED PAY**

0) Salary is based on one's own work: number of patients, interventions, hours worked etc.
1) Salary is based on group work: number of patients, interventions, hours worked etc.
2) Fixed pay, irrespective of the amount of work.

N	Mean	Standard deviation	Median	Interquartile range
109	2.0	0	2.0	0

D2 = GU2 **INADEQUATE PAY**

0) Covers substantially more than my basic needs and those of my family.
0.5) Covers a bit more than my basic needs and those of my family.
1.5) Just barely covers my basic needs and those of my family.
2) Totally inadequate to meet my basic needs and those of my family.

N	Mean	Standard Deviation	Median	Interquartile range
109	0.56	0.55	0.5	0.5

D3: **Possibilities for upgrade**

	Count	% Valid
No	22	20.2
Yes	87	79.8

D3: ***If yes,* is there support and encouragement to do so**?

0) Definitely yes.
0.5) Yes, to some extent.
1) Not really, but there is no active opposition to such efforts.
1.5) No, there is active opposition to such efforts.
2) No, this is viewed very unfavorably and inevitably arouses negative comments or jealousy

N	Mean	Standard Deviation	Median	Interquartile range
86	0.39	0.39	0.5	0.5

D4 = GU4 **LACK OF RECOGNITION OF GOOD WORK**

0) Definitely yes
0.5) Yes, to some extent.
1.5) Not very much.
2) Not at all.

N	Mean	Standard Deviation	Median	Interquartile range
109	0.38	0.46	0.5	0.5

==============================

E. WORKING CONDITIONS

==============================

E1= INOX1 **GLARE EXPOSURE**

0) Never
0.5) Rarely
1) Occasionally (in the Operating Room or elsewhere)
2) Frequently (in the Operating Room or elsewhere)

N	Mean	Standard Deviation	Median	Interquartile range
109	0.61	0.59	0.5	1.0

E2= IAVOI2 **ENCOUNTERS VISUALLY DISTURBING SCENES**

0) Never
0.5) Rarely
1) Occasionally
2) Frequently

N	Mean	Standard Deviation	Median	Interquartile range
109	0.92	0.61	1.0	0.5

E3: **Listens to emotionally disturbing accounts**

0) Never
0.5) Rarely
1) Occasionally
2) Frequently

N	Mean	Standard Deviation	Median	Interquartile range
109	1.08	0.63	1.0	1.5

IAVOI3 **LISTENS TO EMOTIONALLY DISTURBING ACCOUNTS**

0) Never
0.5) Rarely
1) Occasionally
2) Frequently

Add 1 point if B4 = 1.5 or 2) (large percentage of patients with end-stage or incurable disease, 0.5 points if B4=1, add 1 point if F3=1.5 or 2 (patient suicide, and 0.5 points if F3=0.5 (heard about patient suicide), to maximum of 2 points

N	Mean	Standard Deviation	Median	Interquartile range
109	1.47	0.66	2.0	1.0

E4 =ONOX1 **HEAVY LIFTING**

2) Regularly must lift patients or other heavy lifting ≥50 kg
1) Yes, but < 50kg
0) No, rarely does any heavy lifting during the workday.

N	Mean	Standard Deviation	Median	Interquartile range
109	0.13	0.49	0.0	0

E5 = ONOX2 **VIBRATION EXPOSURE**

0) Only very rarely or no vibration exposure
0.5) Uses vibrating hand-tools, up to 5 hours/week
1)Uses vibrating hand-tools, > 5 hours/week

N	Mean	Standard Deviation	Median	Interquartile range
109	0.09	0.24	0.0	0

E6 = GNOX1 **HEAT EXPOSURE**

0) It rarely or never gets hotter than 25° C (77° F) at work.
0.5) It rarely or never gets hotter than 30° C (86° F) at work.
1) It occasionally or often gets hotter than 30° C (86° F).

Add 0.5 points if inadequate ventilation (E13)

N	Mean	Standard Deviation	Median	Interquartile range
109	0.39	0.40	0.5	0.5

E7 = GNOX2 **COLD EXPOSURE**

0) It rarely or never gets colder than 20° C (68° F) at work.
0.5) It rarely or never gets colder than 18° C (64° F) at work.
1) The heating system is poor, with temperatures <18° C (64° F).

Add 0.5 points if drafty (E14)

N	Mean	Standard Deviation	Median	Interquartile range
109	0.30	0.35	0.0	0.5

E8 = GNOX3 **EXPOSURE TO FUMES OR DUST**

0=rarely or never
1=at least occasionally

N	Mean	Standard Deviation	Median	Interquartile range
109	0.32	0.47	0.0	1.0

E9: **Radiation exposure**

	Count	% Valid
Yes	44	40.4
No	65	59.6

E9: **Radiation badge for those with exposure?**

	Count	% Valid
No	29	65.9
Yes	15	34.1

E10: **Acute hazards**

E10a: **Threat of violence**

	Count	% Valid
Yes	29	26.6
No	80	73.4

E10b: **Infection risk**

	Count	% Valid
Yes	89	81.7
No	20	18.3

E10c: **Work with flammable materials**

	Count	% Valid
Yes	6	5.5
No	103	94.5

E10e: **Any acute hazard**

	Count	% Valid
≥ 1 acute hazard	96	88.1
No acute hazards	13	11.9

OVAOIT **HAZARDOUS TASK PERFORMANCE**

_1_ E10= No acute hazards reported
_2_ E10 = a-d Acute hazards present
Add 1 for Radiation exposure, E9 to a maximum of 2 points

N	Mean	Standard Deviation	Median	Interquartile range
109	1.92	0.28	2.0	0

E11: **Shares an office**

	Count	% Valid
Yes	91	83.5
No	18	16.5

E11: **Number of persons sharing an office** (among those who share an office)

N	Mean	Standard Deviation	Median	Interquartile range
91	5.23	3.41	4.0	4.0

E11: **Cramped office**

	Count	% Valid
Yes	53	48.6
No	56	51.4

E11: **Own desk for those who share an office**

	Count	% Valid
No	68	79.1
Yes	18	20.9

E11: **Has to look for an empty office to interview or examine patients**

	Count	% Valid
Yes	80	73.4
No	29	26.6

E11: **Percent time in the office**

N	Mean	Standard Deviation	Median	Interquartile range
108	41.0	32.1	40	60

E12: **Window in office**

	Count	% Valid
Windowless office	14	12.8
Yes, but no direct view outside	34	31.2
Yes, direct view outside	61	56.0

GS2: **CONFINED, WINDOWLESS &/OR POORLY VENTILATED WORK AREA**

_____Works in >1 location (E11part IV < 50% in office (v81<50)) subtract 1 point to minimum 0
_0.5_ Spends over 50% of time in a non-confined work area, with a direct window (E12=a) AND (E11 indicates non-confined work area)
_1_ Spends over 50% of time in a non-confined work area, with an indirect window E12=b) AND (E11 indicates non-confined work area)
_1.5_ Non-confined work area without a window E12=c) AND (E11 indicates non-confined work area-shares with at most 1 other colleagues)
_1.5_ Confined or crowded space with an indirect window (E12=b) AND (E11 indicates very crowded work area or 2+ colleagues in an office)
_2_ Windowless and confined/crowded space (E12=c) AND (E11 indicates very crowded work area or 2+ colleagues in an office)
Add 0.5 points if E13=no, inadequate ventilation, to a maximum of 2 points.

N	Mean	Standard Deviation	Median	Interquartile range
109	1.70	0.64	2.0	0

GS3: **LACKS AUTONOMOUS WORKSPACE**

_0_ E11 (Has own desk/workspace and has own office)
_1_ E11 (Has own desk or workspace but shares an office)
_2_ E11 (No autonomous workspace)
Add 1 point to maximum of 2 if (E11) (V80=1) seeks free office space to talk with or examine patients

N	Mean	Standard Deviation	Median	Interquartile range
108	1.68	0.72	2.0	0

=================================

F. MISHAPS AT WORK

================================

F1=GAVOI1 **EXPERIENCED ACCIDENT OR INJURY AT WORK**

_0_ F1 = No _1_ F1 = Yes, if not serious _2_ F1 = Yes, if serious

N	Mean	Standard Deviation	Median	Interquartile range
109	0.24	0.51	0.0	0

F2=GAVOI2 **WITNESSED ACCIDENT OR INJURY AT WORK**

_0_ F2 = d (never heard about or witnessed a serious accident at work)
_0.5_ F2 = c (heard about but never witnessed a serious accident at work)
_1_ F2 = b (witnessed serious accident at work)
_2_ F2 = a (witnessed fatal accident at work)

N	Mean	Standard Deviation	Median	Interquartile range
109	0.21	0.42	0.0	0

F3: **Patient suicide**

_2_ F3 = a Yes, this has happened on several occasions.
_1.5_ F3 = b Yes, I have had one or two such patients.
_0.5_ F3 = c No, but it has happened to colleague(s) with whom I work.
_0_ F3 = d No, it has never happened to me or to colleague(s) with whom I work

N	Mean	Standard Deviation	Median	Interquartile range
109	0.19	0.45	0.0	0

F4: **Suicide among colleagues or staff at work**

Any suicides (attempts or completed) of colleagues or staff at work

	Count	% Valid
Yes	35	32.1
No	74	67.9

If yes, **how many times has a suicide attempt or completed suicide occurred?**

N	Mean	Standard Deviation	Median	Interquartile range
34	1.77	0.74	2	1.0

If yes, **was the person known to you?**

	Count	% Valid
Yes	22	62.9
No	13	37.1

If yes, **did you work directly with the person or persons?**

	Count	% Valid
Yes	10	29.4
No	24	70.6

If yes, **did any of these result in an actual suicide?**

	Count	% Valid
Yes	23	67.6
No	11	32.4

GAVOI4: **SUICIDE AT WORK (Patient or Other persons at work)**

_0_ F3 = d AND F4 = no: No patient suicide attempt for respondent nor to colleagues. No suicide attempt among colleagues or staff.
_0.5_ F3 = c Patient suicide attempt to colleagues, but not to respondent. No suicide attempt among colleagues or staff.
_1_ F3 = c Patient completed suicide to colleagues, but not to respondent OR F4 = One suicide attempt at work (V93=1), person not known to respondent.
_1.5_ F3= b One or two of respondent's patients have attempted suicide OR F4 >1 suicide attempt at work, persons not known to respondent
_2_ F3 = a Several of respondent's patients have attempted suicide (V90=2) OR F4 Suicide attempt at work with person known to respondent.

Add 1 point to 2 maximum if ANY completed suicide.

N	Mean	Standard Deviation	Median	Interquartile range
109	0.72	0.91	0.0	2.0

F5: **Has testified in court as a physician**

	Count	% Valid
Yes	15	13.8
No	94	86.2

F6: **Court proceedings publicized on mass media**

	Count	% Valid
Yes	9	8.3
No	100	91.7

GAVOI3 **TESTIFYING/LITIGATION/COMPLAINTS**

_0_ F5 = No
_1_ F5 = Yes, As expert witness,
_1.5_ F5 = Yes, Testified about a colleague or staff member or official complaint to workplace
_2_ F5 = Yes, Defendant in a malpractice case

Add 0.5 points if publicized on mass media or if a complaint brought to higher body to maximum 2 points.

N	Mean	Standard Deviation	Median	Interquartile range
109	0.22	0.44	0	0

F10 = GAVOI5 **SYSTEM IN PLACE AT WORK IN CASE OF NON-MEDICAL EMERGENCIES**

0) Yes, and I know that it functions properly.
1) Yes, but I do not know how well it actually functions.
2) No, there is not a functioning system in place in case of non-medical emergencies.

Code	Count	% Valid
2	28	25.7
1	57	52.3
0	24	22.0

==

G. TIME PRESSURE AT WORK

==

G1 =GEP1 **DEADLINE PRESSURE**

Do you have a deadline by which a given job or task must be completed?

0) Never 1) Occasionally
0.5) Rarely 2) Frequently

N	Mean	Standard Deviation	Median	Interquartile range
109	1.48	0.60	2.0	1.0

GEP2: **SPEED-UP WITH EMERGENCY WORK TAKEN INTO ACCOUNT**

0 G2=a (rarely or never) 1 G2=b (certain periods of the month or year)
1.5 G2=c (at least weekly but not daily) 2 G2=d (daily)

Add 0.5 points if B5=c, add 0.25 points if B5=b to a maximum of 2, to account for emergency work.

N	Mean	Standard Deviation	Median	Interquartile range
109	1.49	0.50	1.5	1.0

G3: **With regard to your workload and time constraints**:

0) It is always possible to complete everything.
0.5) It is usually possible to complete everything.
1) It is sometimes impossible to complete everything, even with maximal effort.
2) It is often objectively impossible to complete everything, even with maximal effort.

N	Mean	Standard Deviation	Median	Interquartile range
109	0.77	0.38	0.5	0.5

OCNFL1**: CONFLICTING DEMANDS IN TIME AND SPACE**

0 G3 It is always possible to complete everything.
0.5 G3 It is usually possible to complete everything.
1 G3 It is sometimes impossible to complete everything, even with maximal effort.
2 G3 It is often objectively impossible to complete everything, even with maximal effort.

Add 0.5 points for J14 =no special time set aside for non-clinical tasks, J7= cares for newly admitted patients during shift, to max. 2 points

N	Mean	Standard Deviation	Median	Interquartile range
109	1.56	0.43	1.7	0.5

H. PROBLEMS/RESTRICTIONS/CONSTAINTS & INFLUENCE AT WORK

The degree of influence over:	Major (0)	Some (0.5)	Little (1.5)	None (2)
H1. Number of patients under your care/ outpatient scheduling				
H2. Which clinical tasks or procedures you perform				
H3. Whether and how much you will take on other, non-clinical duties				
H4. Which colleagues and staff you work with				
H5. Planning and policy of your institution (including those regarding indications for medical procedures and for hospital admissions)				

H1: **Influence over number of patients under your care/outpatient scheduling**

N	Mean	Standard Deviation	Median	Interquartile range
109	0.86	0.72	0.5	1.5

H2 : **Clinical task choice influence**

N	Mean	Standard Deviation	Median	Interquartile range
109	0.66	0.68	0.5	1.5

H3 **Non-clinical task choice influence**

N	Mean	Standard Deviation	Median	Interquartile range
109	0.99	0.66	0.5	1.0

H4 **Colleagues & staff choice influence**

N	Mean	Standard Deviation	Median	Interquartile range
109	0.85	0.67	0.5	1.0

H5 = GS8 **PLANNING/POLICY INFLUENCE**

N	Mean	Standard Deviation	Median	Interquartile range
109	1.27	0.72	1.5	1.5

H7. **Is your clinical judgment questioned?**

2) Often
1) Sometimes
0.5) Rarely
0) Never

N	Mean	Standard Deviation	Median	Interquartile range
109	0.81	0.54	1.0	0.5

The degree of strictness of rules and regulations regarding:

	Very strict(2)	Somewhat strict (1)	Flexible (0)	Not applicable
H8. Patient admissions to hospital				
H9. Patient scheduling				

H8. **Strictness regarding patient admissions to hospital**

N	Mean	Standard Deviation	Median	Interquartile range
79	0.52	0.64	0.0	1.0

H9. **Strictness regarding patient scheduling**

N	Mean	Standard Deviation	Median	Interquartile range
81	0.56	0.65	0.0	1.0

H10 = OCNFL2 **TECHNICAL PROBLEMS HAMPERING PATIENT CARE**

0) a) Never
0.5) b) Rarely
1) c) Occasionally
2) d) Frequently

N	Mean	Standard Deviation	Median	Interquartile range
109	0.74	0.36	0.5	0.5

***Specified technical problems*:**

H10a: **Lack of supplies**

	Count	% Valid
Yes	45	41.3

H10b: **Lack of hospital beds**

	Count	% Valid
Yes	37	33.9

H10c: **Understaffing**

	Count	% Valid
Yes	46	42.2

H10d: **Administrative constraints in ordering supplies**

	Count	% Valid
Yes	23	21.1

H103e: **Language barriers**

	Count	% Valid
Yes	50	45.9

H103f: **Infrastructural problems**

	Count	% Valid
Yes	12	11.0

H103g: **Tenuous patient transport**

	Count	% Valid
Yes	9	8.3

H103h: **Delay or inability to obtain medical records**

	Count	% Valid
Yes	19	17.4

H103i: **Difficulty in obtaining laboratory results**

	Count	% Valid
Yes	34	31.5

H103j: **Limitations in ordering tests**

	Count	% Valid
Yes	28	25.7

H10l: **Limitations in patient consults**

	Count	% Valid
Yes	17	15.6%

H11 = OCNFL3 **INTERRUPTIONS FROM PEOPLE HAMPER TASK PERFORMANCE**

0) Never
0.5) Rarely
1) Occasionally
2) Frequently

N	Mean	Standard Deviation	Median	Interquartile range
109	0.79	0.55	0.5	0.5

I. INTERPERSONAL INTERACTIONS & SOCIAL CLIMATE

I1: GCNFL2 **LACKS HELP WITH CLINICAL DIFFICULTIES**

Can you get help for difficult cases and/or clinical dilemmas?

0) Yes, I can almost always count on such help.
0.5) Yes, more often than not
1.5) I can't really count on getting such help.
2) Rarely or never do I get the help, which I need.

N	Mean	Standard Deviation	Median	Interquartile range
109	0.16	0.26	0.0	0.5

I2**: Social climate: In general, how is the social climate at your workplace**?

0) Excellent, we all get along very well together and misunderstanding are very rare.
0.5) Good, most the time we get along well, with few misunderstandings and tensions.
1) Fair, we have our ups-and-downs, and sometimes there are misunderstandings and tensions.
2) Poor, there is a great deal of tension and conflict.

N	Mean	Standard Deviation	Median	Interquartile range
109	0.43	0.38	0.5	0.5

I3: **Knowledge display atmosphere: When obliged to display knowledge and/or skills in front of colleagues and/or supervisors (e.g. at rounds, journal club, other presentations):**

0) The atmosphere is constructive and conducive to growth and learning
0.5) There is some tension. Oversights and/or lack of knowledge will be noticed and commented upon. If these are of major importance, there may be adverse consequences for me.
1) These occasions are highly unpleasant. Even the slightest oversight or lack of knowledge inevitably becomes a point of ridicule and/or chastisement.

N	Mean	Standard Deviation	Median	Interquartile range
109	0.15	0.24	0.0	0.5

GCNFL1: **EMOTIONALLY CHARGED WORK ATMOSPHERE**

0 I2 = (no) AND I3=0 (Constructive learning atmosphere)
0.5 I2 =0.5
1 I2 =1 (occasionally)
2 I2 =2 (great deal of tension)

Add 0.5 points if I3 = 0.5) (some tension with display of knowledge) and 1 point if I3=1 (high tension with display of knowledge) to a maximum of 2 points.

N	Mean	Standard Deviation	Median	Interquartile range
109	0.57	0.53	0.5	1.0

I4= GCNFL4 **ABUSE OF POWER/VIOLATIONS OF NORMS OF BEHAVIOR**

(for example, blocking career development, discrimination, mobbing, sexual harassment)

0) Never 0.5) Rarely 1.5) Occasionally 2) Frequently

N	Mean	Standard Deviation	Median	Interquartile range
109	0.17	0.43	0.0	0

I5=GCNFL5: **CAN WORK-RELATED GRIEVANCES BE TAKEN TO A RESPONSIBLE BODY FOR RESOLUTION**?

0) Yes, this can be done in an efficient and confidential manner.
1) In principle, yes, but this is not effective and/or cannot be done confidentially.
2) No, there is no possibility to redress grievances at work.

N	Mean	Standard Deviation	Median	Interquartile range
109	0.59	0.64	1.0	1.0

=============================

J. WORKLOAD & ACTIVITIES

=============================

J1: **Handles patients who are severely disturbed**

Frequently (2), Occasionally (1) Rarely/Never (0)

N	Mean	Standard Deviation	Median	Interquartile range
109	1.12	0.65	1.0	1.0

J2: **Handles patients who cannot give a history**

Frequently (2), Occasionally (1) Rarely/Never (0)

N	Mean	Standard Deviation	Median	Interquartile range
109	1.12	0.65	1.0	1.0

J3: **Number of inpatients under the physician's direct care at one time**

0) None
1.5) One to five
2) Six to ten
3) Eleven to twenty
4) Over twenty

N	Mean	Standard Deviation	Median	Interquartile range
102	2.03	1.24	2.0	1.5

J4: **Number of patients in Intensive care unit/coronary care unit**:

0) None
1.5) One to two
2) Three to five
3) Six to ten
4) Over ten

N	Mean	Standard Deviation	Median	Interquartile range
103	0.96	0.98	1.5	1.5

J5: **Number of outpatients per shift**

0) None
1.5) One to ten
2) Eleven to twenty
3) Twenty-one to thirty
4) Thirty-one to forty
5) Over forty

N	Mean	Standard Deviation	Median	Interquartile range
109	3.11	1.64	3.0	3.0

J6: **Number of hospital admissions per shift**:

0) a) None
1.5) b) One to five
2) c) Six to ten
3) d) Eleven to twenty
4) e) Over twenty

N	Mean	Standard Deviation	Median	Interquartile range
109	1.24	0.84	1.5	1.5

J7: *If you admit patients to the hospital,* **are you obliged to care for these newly-admitted patients, or do you transfer them to other colleagues**?

2) I must care for all the patients whom I admit during a given shift.
1.5) I must care for some of the patients whom I admit during a given shift.
0) The patients whom I admit are nearly always transferred fairly rapidly to other colleagues.

	Count	% Valid
2	38	46.9
1.5	34	42.0
0	9	11.1

J8: **Simultaneous attention: Does it happen during your workday that several people seek your attention at the same time?** *(Including on the telephone)*

2) Yes, many times each day.
1.5) Yes, a few times each day.
1) Yes, but only once or twice each day.
0) No, rarely or never.

N	Mean	Standard Deviation	Median	Interquartile range
109	1.52	0.56	1.5	0.5

J9. **If people simultaneously seek your attention, how many do so**?

0) Rarely more than two 1) Usually two, but sometimes more 2) Usually three or more

N	Mean	Standard Deviation	Median	Interquartile range
109	1.01	0.67	1.0	0

J10=OU3 **DURING WORK HOURS, MUST BE PHYSICALLY AT WORK, BUT THERE IS NOTHING TO DO**

Include time when waiting and cannot proceed with other work

0) Never 0.5) Rarely 1) Occasionally 2) Frequently

N	Mean	Standard Deviation	Median	Interquartile range
109	0.58	0.47	0.5	0.5

Supervising

J11a: **Supervises physicians at same or higher level**

	Count	% Valid
Yes	21	19.3

J11b: **Supervises physicians with less training**

	Count	% Valid
Yes	72	66.1

J11c: **Supervises medical students**

	Count	% Valid
Yes	77	70.6

J11d: **Supervises other health professionals**

	Count	% Valid
Yes	45	41.3

J12: **Number supervised**

N	Mean	Standard Deviation	Median	Interquartile range
109	3.73	3.17	3.0	3.0

CH3 (supervising): **DECISIONS AFFECT THE WORK OF OTHERS**

1 J11-J12=no direct supervision (but some always implicit for physicians)
1.5 J11-J12=supervises one to two others
2 J11-J12 =supervises three or more others

N	Mean	Standard Deviation	Median	Interquartile range
109	1.71	0.33	2.0	0.5

J13: **Other duties besides clinical work**

	Count	% Valid
Yes	109	100

J13a: **Teaching in small groups**

	Count	% Valid
Yes	91	83.5

J13b: **Lecturing to larger groups**

	Count	% Valid
Yes	30	27.5

J13c: **Research**

	Count	% Valid
Yes	79	(72.5

J13d: **Administrative duties**

	Count	% Valid
Yes	38	34.9

J14:: **Separate time allocated for non-clinical duties**

	Count	% Valid
No	81	74.3
Yes	28	25.7

If no separate time, when are these other duties performed?

J14a:: **Interspersed with clinical work**

	Count	% Valid
Yes	64	80%

J14b:: **Outside work hours**

	Count	% Valid
Yes	45	56.3

================

J15: **Publishing pressure Are you under pressure to publish/present new findings or results at Congresses/meetings outside your Institution?**

0.5) Yes, if I fail to do so in sufficient quantity, my career will suffer and I may even lose my position
0.25) Yes, but there are no major adverse consequences if I fail to do so.
0) No, such activity is entirely up to my own initiative and choice.

N	Mean	Standard Deviation	Median	Interquartile range
109	0.21	0.20	0.25	0.5

Task specific queries:

J16: **Performs non-invasive diagnostic procedures**

	Count	% Valid
Yes	95	87.2

J17: **Performs invasive procedures**

	Count	% Valid
Yes	86	78.9

J18: **Performs surgical interventions**

	Count	% Valid
Yes	55	50.5

J19: **Performs tasks outside the realm of a physician/work of other personnel**

	Count	% Valid
Yes	32	29.4

J20-GCNFL7 **PERFORMS TASKS THAT SEEM POINTLESS**

	Count	% Valid
Yes	26	23.9

J21: **Regularly uses the computer for clinical work**

	Count	% Valid
Yes	109	100

For patient write-ups

	Count	% Valid
Yes	86	78.9

For triage

	Count	% Valid
Yes	36	33.0

For statistical analyses that would be for clinical purposes

	Count	% Valid
Yes	52	47.7

For electronic communication with colleagues (e.g. consults)

	Count	% Valid
Yes	74	67.9

For communication with patients

	Count	% Valid
Yes	17	15.6

For searching the medical literature to elucidate a clinical question

	Count	% Valid
Yes	90	82.6

==

K. RECENT CHANGES AT WORK

==

K1: **An increase in workday length**

	Count	% Valid
Yes	62	56.9

K2: **An increase in time pressure/deadlines**

	Count	% Valid
Yes	46	42.2

K3: **An increase in responsibility**

	Count	% Valid
Yes	89	81.7

K6 **Recent promotion**

	Count	% Valid
Yes	16	14.7

Acronyms and Abbreviations

AVOI	Symbolic aversiveness/avoidance/disaster potential (OSI aspect)	NOX	Noxious physical exposures (OSI aspect)
C	Central decision making (OSI level)	O	Output (OSI level)
CNFL	Conflict/uncertainty (OSI aspect)	OSI	Occupational Stressor Index
EP	Extrinsic time pressure (OSI aspect)	S	Strictness (OSI aspect)
G	General (OSI level)	T	Total
H	High demand (OSI aspect)	U	Underload (OSI aspect)
I	Input (OSI level)		
